# Supplementary material for: Phonons in Short-Period GaN/AlN Superlattices: Group-Theoretical Analysis, Ab initio Calculations, and Raman Spectra
Source: Nanomaterials (Basel). 2021 Jan 22;11(2):286. doi: 10.3390/nano11020286 (PMC7911830; doi:10.3390/nano11020286)
Supplement: Supplementary file 1 [file nanomaterials-11-00286-s001.pdf]

nanomaterials

Supplementary

Phonons in short-period GaN/AlN superlattices: group-theoretical analysis, *ab initio* calculations, and Raman spectra

Valery Davydov *, Evgenii Roginskii, Yuri Kitaev, Alexander Smirnov, Ilya Eliseyev, Dmitrii Nechaev, Valentin Jmerik, Mikhail Smirnov

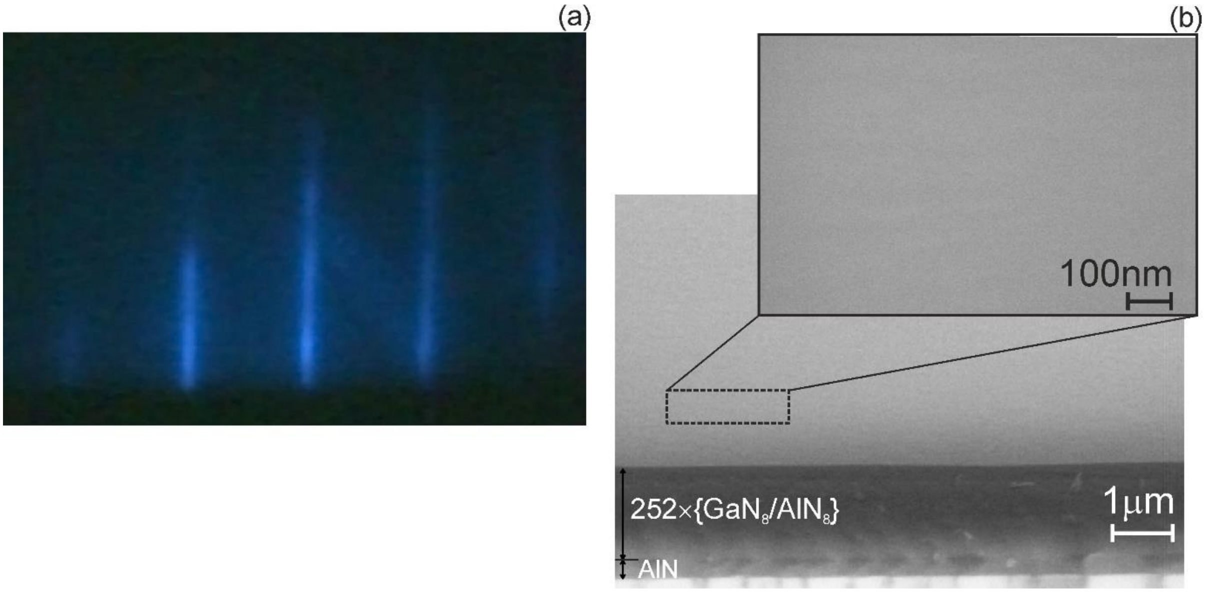

Figure S1. RHEED pattern observed during the growth (**a**) and SEM images with different magnification of 42×6×{$(GaN)_8/(AlN)_8$} SL studied in this article (**b**).

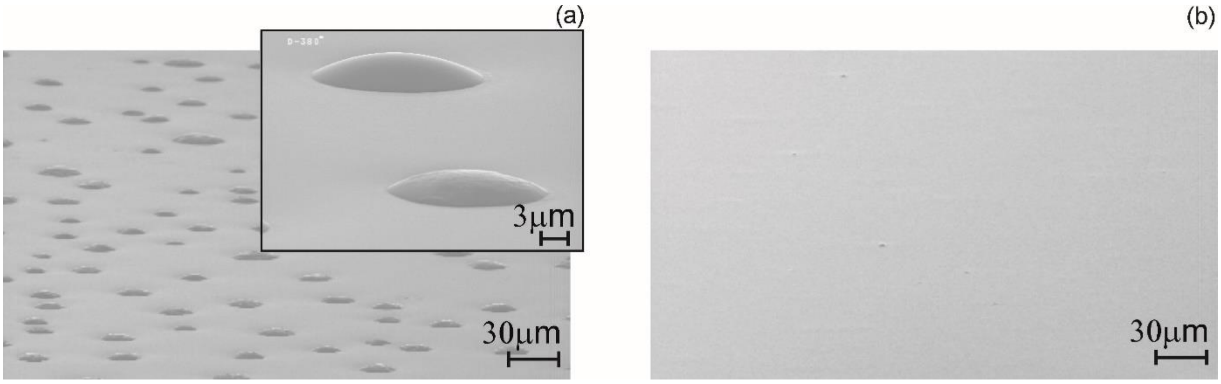

Figure S2. SEM images with different magnifications of two SLs grown under different growth conditions providing 2D-droplet surface morphology (**a**) and 2D-droplet-free morphology, as in 42×6×{$(GaN)_8/(AlN)_8$} studied in this article (**b**).

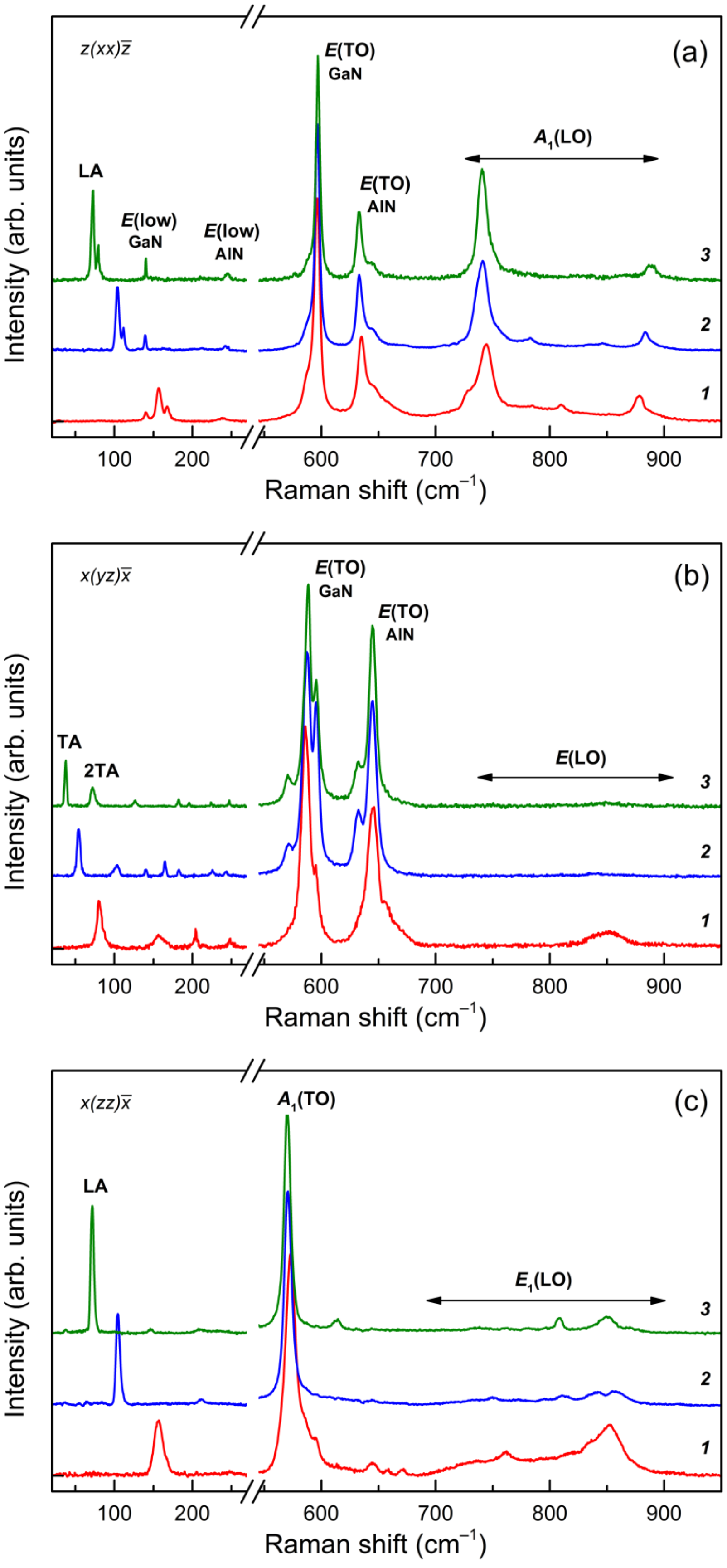

Figure S3. Experimental Raman spectra in $z(xx)\bar{z}$ (**a**), $x(yz)\bar{x}$ (**b**), and $x(zz)\bar{x}$ (**c**) scattering geometries for the $(GaN)_m/(AlN)_n$ SLs with m/n= 4/4 (*1*), 6/6 (*2*), and 8/8 (3).
